# Six-months clinical and intracoronary imaging follow-up after reverse T and protrusion or double-kissing and crush stenting for the treatment of complex left main bifurcation lesions

**DOI:** 10.3389/fcvm.2023.1153652

**Published:** 2023-04-27

**Authors:** Amr EI Abouelnour, Maximilian Olschewski, Giulio Makmur, Helen Ullrich, Maike Knorr, Majid Ahoopai, Thomas Münzel, Tommaso Gori

**Affiliations:** ^1^ University Medical Center Mainz, Department of Cardiology, Mainz, Germany and German Center for Cardiac and Vascular Research (DZHK), Standort Rhein-Main, Germany; ^2^ Department of Cardiovascular Medicine, Assiut University Heart Hospital, Assiut, Egypt

**Keywords:** left main, bifurcation, optical coherence tomography (OCT), reverse t and protrusion, double kissing and crush

## Abstract

**Background:**

There is a debate regarding the best stent strategy for unprotected distal left main (LM) bifurcation disease. Among two-stent techniques, double-kissing and crush (DKC) is favored in current guidelines but is complex and requires expertise. Reverse T and Protrusion (rTAP) was shown to be a comparable strategy regarding short-term efficacy and safety, but with reduced procedural complexity.

**Aim:**

To compare rTAP vs. DKC by optical coherence tomography (OCT) on the intermediate term.

**Methods:**

52 consecutive patients with complex unprotected LM stenoses (Medina 0,1,1 or 1,1,1) were randomized to either DKC or rTAP and followed-up for a median of 189[180–263] days for clinical and OCT outcomes.

**Results:**

At follow-up OCT showed similar change in the side branch (SB) ostial area (primary endpoint). The confluence polygon showed a higher percentage of malapposed stent struts in the rTAP group that did not reach statistical significance (rTAP: 9.7[4.4–18.3] % vs. DKC: 3[0.07–10.9] %; *p* = 0.064). It also showed a trend towards larger neointimal area relative to the stent area (DKC: 8.8 [6.9 to 13.4] % vs. rTAP: 6.5 [3.9 to 8.9] %; *p* = 0.07), and smaller luminal area (DKC: 9.54[8.09–11.07] mm^2^ vs. rTAP: 11.21[9.53–12.42] mm²; *p* = 0.09) in the DKC group. The minimum luminal area in the parent vessel distal to the bifurcation was significantly smaller in the DKC group (DKC: 4.64 [3.64 to 5.34] mm² vs. rTAP: 6.76 [5.20 to 7.29] mm²; *p* = 0.03). This segment also showed a trend for smaller stent areas (*p* = 0.05 to 0.09), and a bigger neointimal area relative to the stent area (DKC: 8.94 [5.43 to 10.5]% vs. rTAP: 4.75 [0.08 to 8.5]%; *p* = 0.06) in the DKC patients. The incidence of clinical events was comparably low in both groups.

**Conclusion:**

At 6-months, OCT showed a similar change in the SB ostial area (primary endpoint) in rTAP compared to DKC. There was also a trend for smaller luminal areas in the confluence polygon and the distal parent vessel, and a larger neointimal area relative to the stent area, in DKC, along with a tendency for more malapposed stent struts in rTAP.

**Clinical Trial Registration:**

https://clinicaltrials.gov/ct2/show/NCT03714750, identifier: NCT03714750.

## 1. Introduction

Although distal left main (LM) disease has been treated with coronary artery bypass grafting for several decades, the advances in stent designs, adjunctive pharmacotherapy, and the evolution of intravascular imaging to guide and optimize percutaneous coronary interventions (PCI), make interventional treatment a viable alternative in cases with low to intermediate syntax score (1). In an attempt to improve immediate and long-term clinical outcomes, multiple stenting techniques have been proposed, and disagreement exists on whether an upfront single- or two-stent strategy is superior (2). In cases of severe disease in both branches, where an upfront 2-stent strategy is planned, guidelines recommend the DK-Crush (DKC) technique based on evidence from trials comparing the outcomes of this strategy to classic Crush (3) and Culotte stenting (4, 5). Although the discussion regarding the relative merits of different strategies remains open, 2-stent techniques involve many technical steps and demand expertise (6). Consequently, any efforts at simplifying these processes, including reducing procedural times, contrast and x-ray exposure while preserving patient outcomes are welcome. In a recently published paper, we reported the outcomes of a randomized trial comparing DKC with a simplified variant involving a shorter stent crush length and only one rewiring. We reported that this variation, which we called reverse-T-and-protrusion (rTAP) and which resembles a previously reported technique named “cone flare DKC”(7), was non-inferior to DKC in terms of side branch (SB) ostial expansion and was associated with shortened procedural times (8). In the present study, we compare the intermediate-term outcomes assessed by optical coherence tomography (OCT) of patients with LM bifurcation disease, randomized to either the DKC technique or rTAP. The purpose of the new proposed technique is to minimize procedural time and complexity without compromising patient outcomes.

## 2. Materials and methods

### 2.1. Study design

**Figure 1:**
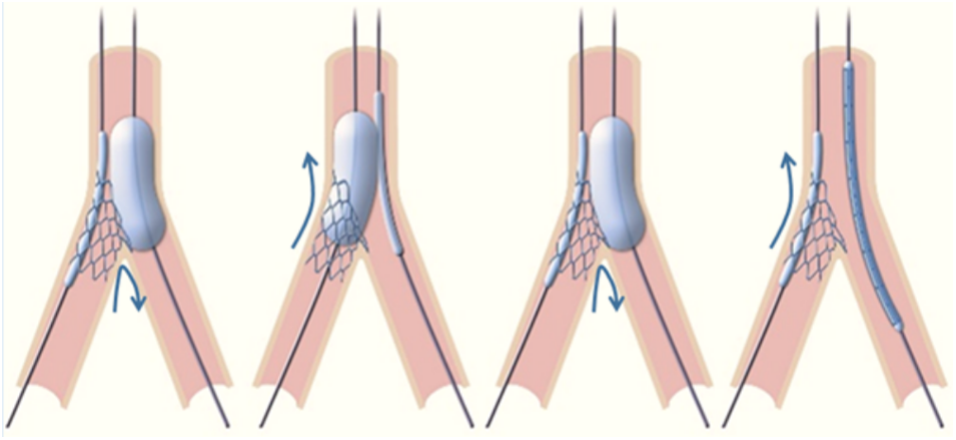
**Reverse TAP alternate high-pressure balloon inflations** as in the “standard” TAP technique, the goal of these dilatations is to progressively flare the proximal SB stent at the ostium, so that the struts are spread across its circumference. As compared to crush techniques, this approach is designed to avoid double layers of struts on one side. The high-pressure dilations are designed to optimize SB ostium expansion.

In this 1:1 randomized controlled trial (NCT03714750), 52 consecutive patients with unprotected complex distal LM bifurcation disease (Medina 0,1,1 or 1,1,1) and a heart team recommendation for PCI treatment were randomly assigned to either of 2 strategies: DKC or rTAP (26 patients in each arm). The inclusion and exclusion criteria as well as the details of both stenting techniques were previously described (9) (Figure 1 depicts the critical step specific to rTAP). Randomization was performed by a computer-generated random sequence (MedCalc Statistical Software version 15.1 (MedCalc Software bvba, Ostend, Belgium)). The study was approved by the local ethics committee and all enrolled patients provided written informed consent.

### 2.2. End points

The primary purpose of the current study is to compare the OCT-assessed change in the SB ostial area between the two study arms at 6-months follow-up. As secondary endpoints, we also compared stent and luminal areas, as well as neointimal areas relative to the respective stent areas, in all segments of the bifurcation, in addition to the percentage of malapposed and uncovered stent struts in the confluence polygon.

#### 2.2.1. Optical coherence tomography (OCT)

Patients were scheduled for follow-up diagnostic coronary angiography at 6 months. OCT was performed using the Dragonfly^TM^ Duo ILUMIEN^TM^ catheter (St. Jude Medical, St. Paul, MN, USA). Two pullbacks were performed from the main branch (MB; parent distal artery), and from the SB to the LM stem, at the speed of 18 mm/second (high resolution mode), to examine the different bifurcation segments. Offline analysis of the acquired data sets was then carried out using QCU-CMS Version 4–69 (Leiden University Medical Center and MEDIS, Leiden, the Netherlands) by a cardiologist blinded to the patient allocation, to the initial post-PCI results as well as to the patients' clinical outcome.

**Figure 2:**
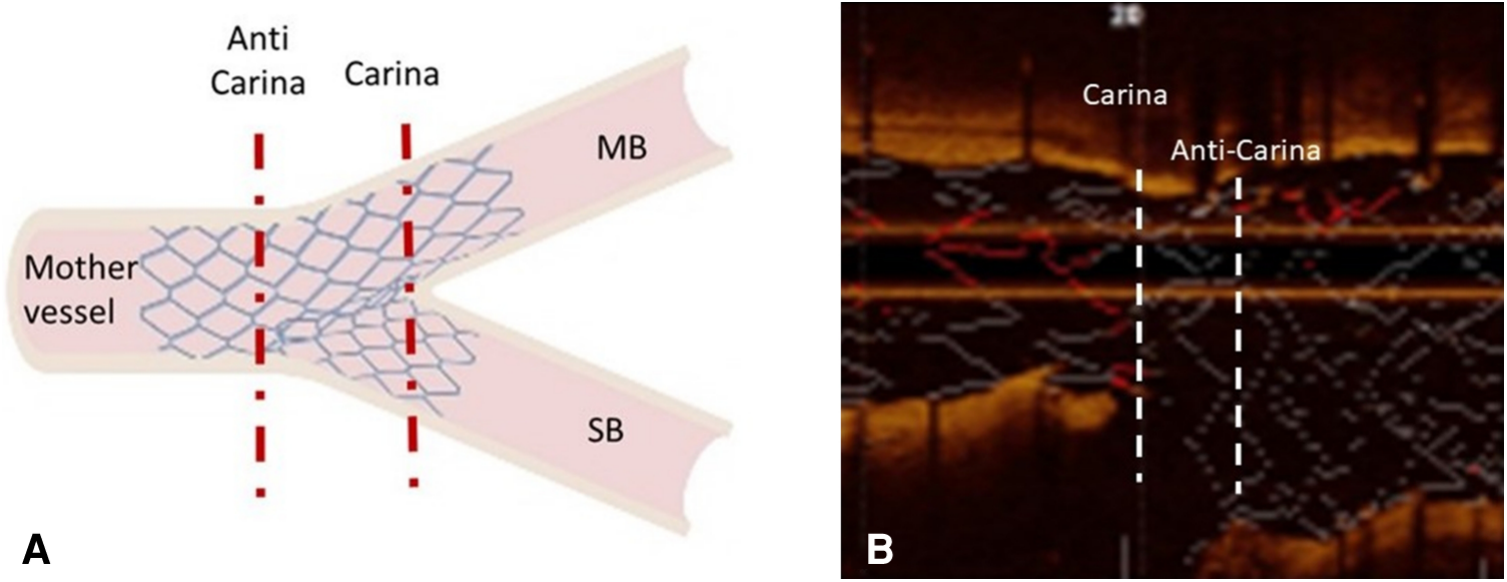
**Confluence polygon (carina-anticarina zone)** (**A**) is a schematic depicting the region of the confluence polygon contained between the two dashed red lines. (**B**) is an example of an OCT longitudinal view showing the confluence polygon confined by the 2 dashed white lines. MB: main branch; SB: side branch.

Analysis was performed at 1-mm intervals except for the so-called confluence polygon (carina-anticarina zone, Figure 2), which was analyzed on a frame-by-frame basis. The cross-sectional images were examined in the confluence polygon for the mean stent area, mean luminal area, absolute mean neointimal area (=mean stent area-mean luminal area). The mean neointimal area normalized to the mean stent area was then derived. In other bifurcation segments, the maximum, and minimum stent areas, as well as the minimum luminal area were noted. The mean neointimal area normalized to the mean stent area was again derived. The stent struts were automatically detected by the software on the frames of interest, and the stent and luminal contours were automatically traced (with manual corrections if needed). The percentages of malapposed and uncovered stent struts were calculated. All malapposed struts were counted regardless of the severity/distance of the malapposition.

The change in the SB ostial area from baseline to follow-up was also calculated.

#### 2.2.2. Clinical end points

Patients were followed-up by office visits or telephone contact for standardized clinical endpoints (10) including: a patient-oriented composite of all-cause mortality, any revascularization, and any myocardial infarction (MI), in addition to a lesion-specific composite of cardiovascular (CV) death, target lesion revascularization (TLR) (non-protocol-driven), and target vessel MI.

### 2.3. Statistical analysis

IBM SPSS Statistics Version 23.0.0.2 (2015) and MedCalc Statistical Software version 15.8 (MedCalc Software bvba, Ostend, Belgium; 2015) were used. Continuous variables are presented as medians with interquartile ranges. Categorical variables are presented as counts and percentages. Mann-Whitney *U* test was used to compare continuous variables between the 2 study arms. A *p* value < 0.05 was considered statistically significant. The Benjamini Hochberg method was used to adjust for multiple comparisons.

## 3. Results

The baseline clinical characteristics, procedural characteristics, and procedural outcomes, as well as OCT analysis results, of the 52 enrolled patients were previously reported (8). There were no differences between groups in any of the clinical, procedural, or imaging variables except for ostial side branch expansion, which was significantly larger in the rTAP at the end of the procedure (8).

### 3.1. Clinical outcomes

Clinical follow-up was available in all patients at 6 months in both groups (Table 1). The lesion-specific endpoint was achieved in only 1 patient in the DKC group (TLR due to unstable angina) and 2 patients in the rTAP group (1 CV death by cardiogenic pulmonary edema, and 1 TLR due to unstable angina). None of the patients had in-stent thrombosis nor STEMI or NSTEMI.

**Table 1:** Clinical outcomes at 6-months follow-up.

**Clinical end-point**	Study arm			
	DK crush		Reverse TAP	
Patient-oriented composite	No	24 (92.3%)	No	22 (84.6%)
	Yes	2 (7.7%)	Yes	4 (15.4%)
Lesion-specific composite	No	25 (96.2%)	No	24 (92.3%)
	Yes	1 (3.8%)	Yes	2 (7.7%)
MACE	No	25 (96.2%)	No	24 (92.3%)
	Yes	1 (3.8%)	Yes	2 (7.7%)
All-cause mortality	No	26 (100%)	No	24 (92.3%)
	Yes	0 (0%)	Yes	2 (7.7%)
Cardiovascular death	No	26 (100%)	No	25 (96.2%)
	Yes	0 (0%)	Yes	1 (3.8%)
Any revascularization	No	24 (92.3%)	No	24 (92.3%)
	Yes	2 (7.7%)	Yes	2 (7.7%)
TVR	No	24 (92.3%)	No	24 (92.3%)
	Yes	2 (7.7%)	Yes	2 (7.7%)
Non-target vessel revascularization	No	25 (96.2%)	No	26 (100%)
	Yes	1 (3.8%)	Yes	0 (0%)
TLR	No	25 (96.2%)	No	25 (96.2%)
	Yes	1 (3.8%)	Yes	1 (3.8%)
TVF	No	25 (96.2%)	No	24 (92.3%)
	Yes	1 (3.8%)	Yes	2 (7.7%)
Any MI	No	26 (100%)	No	26 (100%)
	Yes	0 (0%)	Yes	0 (0%)
Target vessel MI	No	26 (100%)	No	26 (100%)
	Yes	0 (0%)	Yes	0 (0%)
Target vessel STEMI	No	26 (100%)	No	26 (100%)
	Yes	0 (0%)	Yes	0 (0%)
Target vessel NSTEMI	No	26 (100%)	No	26 (100%)
	Yes	0 (0%)	Yes	0 (0%)
In-stent thrombosis	No	26 (100%)	No	26 (100%)
	Yes	0 (0%)	Yes	0 (0%)

Events expressed as count (% of total cases in a study arm).

The patient-oriented endpoint was achieved in 2 patients (7.7%) in the DKC arm, who received revascularization vs. 4 patients (15.4%) in the rTAP arm (2 deaths and 2 patients who received revascularization). The Two deaths in the rTAP group included 1 CV death (cardiogenic pulmonary edema at 147 days), and 1 non-CV death (prostatic cancer at 77 days).

Per-protocol coronary angiography was performed in 24 patients at a median of 188.5 days (133 to 337) after index PCI in the DKC group and in 21 patients at a median of 181 days (113 to 378) in the rTAP group. Four patients refused the control coronary angiography, and three others died before scheduled control. None of these patients had symptoms or non-invasive evidence of ischemia.

### 3.2. OCT analysis results

Of the 45 patients with control coronary angiography, OCT was available in 38 patients (except for SB pullback in a single patient where the OCT catheter failed to cross into the SB).

Baseline clinical characteristics of the patients with follow-up OCT are reported in Supplementary Table S1, and procedural details are reported in Supplementary Table S2. Pre-defined protocol and procedural success was achieved in all patients in both arms (definitions previously reported (8, 9)), with no difference in peak post-procedural troponin between the 2 arms.

#### 3.2.1. Change in side branch ostial area

**Figure 3:**
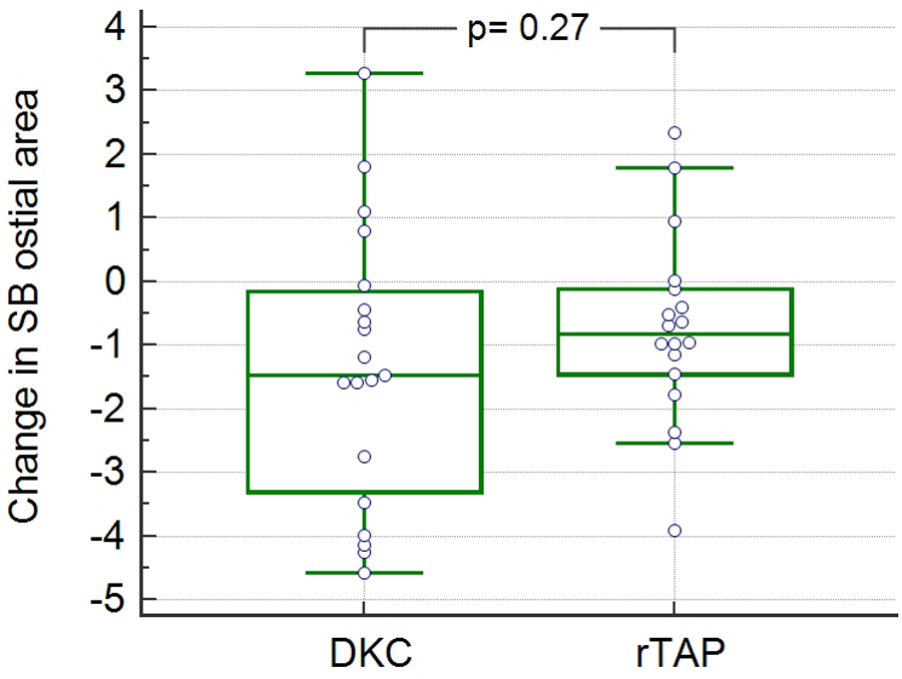
**Change in side branch ostial area by OCT in the two study groups** OCT: optical coherence tomography; SB: side branch; DKC: double kissing and crush; rTAP: reverse T and protrusion.

No significant difference was found regarding the change in the SB ostial area from baseline to follow-up in the 2 groups (−1.48[−3.3 to −0.15] mm^2^ in DKC vs. −0.82[−1.45 to −0.12] mm^2^ in rTAP; *p* = 0.38) (Table 3, Figure 3).

#### 3.2.2. Confluence polygon analysis results

**Figure 4:**
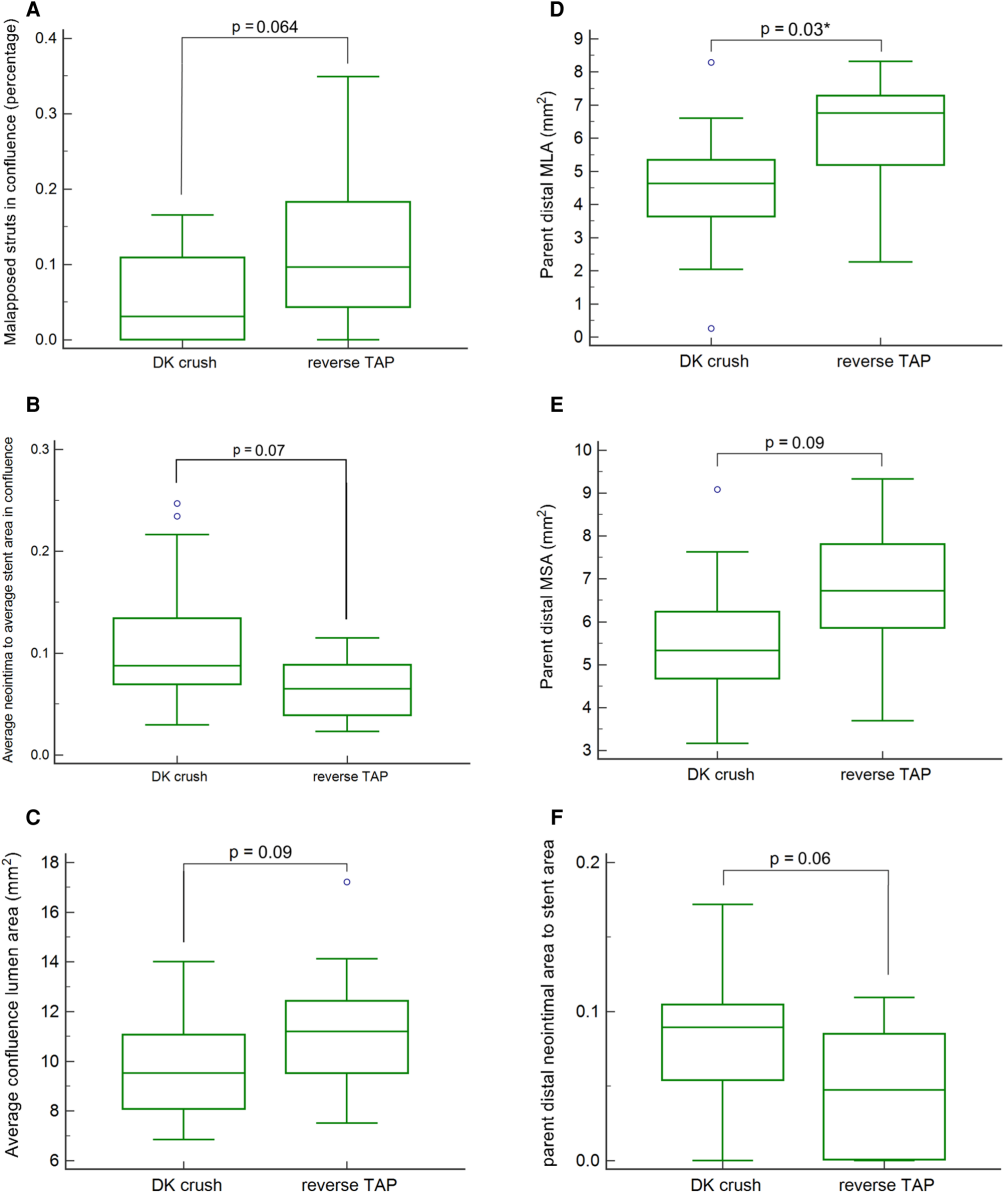
(**A**) malapposed stent strut percentage in the confluence polygon in the two study groups. (**B**). Mean neointimal area normalized to mean stent area in the confluence polygon in the two study groups. (**C**) Mean luminal area in the confluence polygon in the two study groups. (**D**) Minimal luminal area (MLA) in the main branch distal to the bifurcation in the two study groups *statistically significant. (**E)** Minimal stent area (MSA) in the main branch distal to the bifurcation in the two study groups. (**F**) Mean neointimal area normalized to mean stent area in the main branch in the two study groups.

There was a trend for a higher percentage of malapposed stent struts in the rTAP group (rTAP: 9.7 [4.4–18.3] % vs. DKC: 3 [0.07–10.9] %; *p* = 0.064) that did not reach statistical significance (Figure 4A). However, there was no difference regarding the percentage of uncovered struts (*p* = 0.22) (Table 2).

**Table 2:** Confluence polygon OCT analysis results.

** **	DK Crush			** **	Reverse TAP	** **	*p*	Adjusted p
	Median	IQR	Average rank	Median	IQR	Average rank		
Number of struts	227	143 to 365	19.34	275	182 to 331	19.66	0.93	1.01
Malapposed struts (%)	3	0.07 to 10.9	15.16	9.7	4.4 to 18.3	23.84	0.016	0.064*
Uncovered struts (%)	1.3	0.08 to 4	16.55	2.4	1.3 to 6.8	22.45	0.10	0.22
Mean stent area (mm^2^)	10.53	9.41 to 11.92	16.84	12.01	10.40 to 12.92	22.16	0.14	0.24
Mean lumen area (mm^2^)	9.54	8.09 to 11.07	15.63	11.21	9.53 to 12.42	23.37	0.032	0.09*
Mean neointimal area/mean stent area (%)	8.8	6.9 to 13.4	23.89	6.5	3.9 to 8.9	15.11	0.015	0.07*

* Adjusted *p* < 0.1. IQR: inter-quartile range.

There was also a non-statistically significant trend towards larger neointimal area relative to the stent area (DKC: 8.8 [6.9 to 13.4] % vs. rTAP: 6.5 [3.9 to 8.9] %; *p* = 0.07), and smaller luminal area (DKC: 9.54[8.09–11.07]mm^2^ vs. rTAP: 11.21[9.53–12.42]mm²; *p* = 0.09) in the DKC group (Figures 4B, C respectively), despite no difference in the stent area (10.53 [9.41 to 11.92] mm^2^ in DKC vs. 12.01 [10.4 to 12.92] mm^2^ in rTAP; *p* = 0.2).

#### 3.2.3. Other bifurcation segment analysis results

The minimum luminal area in the parent vessel distal to the bifurcation was significantly smaller in the DKC group (DKC: 4.64 [3.64 to 5.34] mm² vs. rTAP: 6.76 [5.20 to 7.29] mm²; *p* = 0.03) (Figure 4D). This segment also showed a trend for smaller stent areas (*p* = 0.05 to 0.09), and a bigger neointimal area relative to the stent area (DKC: 8.94 [5.43 to 10.5] mm^2^ vs. rTAP: 4.75 [0.08 to 8.5] mm^2^; *p* = 0.06) in the DKC patients (Figures 4E, F respectively) (Table 3).

**Table 3:** Other bifurcation segment OCT analysis results.

	DK Crush			Reverse TAP			*p*	Adjusted p
	Median	IQR	Avge rank	Median	IQR	Avge rank		
***Mother vessel***								
Maximum stent area [mm²]	11.03	10.09 to 13.85	16.63	13.380	11.60 to 15.77	22.37	0.11	0.2
Minimum stent area [mm²]	8.91	7.81 to 10.52	17.11	10.57	8.14 to 11.50	21.89	0.18	0.27
Minimum lumen area [mm²]	7.48	6.69 to 8.75	15.32	9.82	8.63 to 11.13	23.68	0.02	0.06
Average neointimal to stent area [%]	9.27	3.05 to 15.3	22.05	5.64	1.04 to 8.78	16.95	0.16	0.26
***Main branch***								
Maximum stent area [mm²]	8.18	7.27 to 8.58	14.79	10.13	8.93 to 11.08	24.21	0.009	0.054
Minimum stent area [mm²]	5.33	4.68 to 6.24	15.74	6.73	5.86 to 7.82	23.26	0.037	0.09
Minimum lumen area [mm²]	4.64	3.64 to 5.34	14.37	6.76	5.20 to 7.29	24.63	0.004	0.03*
Average neointimal to stent area [%]	8.94	5.43 to 10.5	23.76	4.75	0.08 to 8.5	15.24	0.018	0.06
***Side branch***								
Maximum stent area [mm²]	8.29	7.33 to 9.93	19.74	8.94	5.82 to 9.24	18.22	0.67	0.8
Minimum stent area [mm²]	5.36	4.07 to 6.04	19.76	4.58	3.80 to 6.43	18.19	0.66	0.83
Minimum lumen area [mm²]	3.79	3.05 to 5.70	18.03	4.13	3.43 to 5.82	20.03	0.57	0.8
Average neointimal to stent area [%]	12.4	6.43 to 15.7	21.84	8.8	4 to 12.4	16	0.1	0.2
***Main branch ostium***								
Stent area [mm²]	6.15	5.56 to 7.45	13.95	8.05	6.98 to 9.03	25.05	0.002	0.02*
Lumen area [mm^2^]	5.3	4.66 to 6.62	12.84	8.03	6.87 to 9.63	26.16	0.0002	0.005*
***Side branch ostium***								
Stent area[mm²]	5.76	4.80 to 6.86	19.42	5.75	4.46 to 7.07	18.56	0.81	0.93
Lumen area [mm^2^]	4.70	3.78 to 6.33	19.11	4.74	3.98 to 5.86	18.89	0.95	0.99
Change in SB ostial area (mm^2^)	−1.48	−3.3 to −0.15	17.11	−0.82	−1.45 to −0.12	21	0.27	0.38

* Statistically significant. IQR: inter-quartile range; Avge: average.

The main branch ostium had a significantly smaller stent and luminal area in the DKC group (6.15 [5.56 to 7.45] mm^2^ in DKC vs. 8.05 [6.98 to 9.03] mm^2^ in rTAP; *p* = 0.02, and 5.3 [4.66 to 6.62] mm^2^ in DKC vs. 8.03 [6.87 to 9.63] mm^2^ in rTAP; *p* = 0.004, respectively).

There was no significant difference in the SB stent or luminal areas between the 2 study groups.

## 4. Discussion

The differences between rTAP and DKC have been extensively described in a previous paper and are summarized in Figure 1 (8). The major differences include the use of a systematic alternate high-pressure inflation of MB and SB balloons following SB stent implantation and the need for one rewiring in rTAP (instead of two in DKC). In rTAP, this alternate balloon dilation results in the splaying of the proximal SB stent struts along the circumference of the SB ostium. In DKC, the first MB balloon inflation followed by the first rewiring and kissing results in crushing of the proximal SB stent struts on one side.

In our previous publication, the rTAP technique proved to be non-inferior to the gold standard DKC technique in terms of short-term efficacy and safety in the treatment of complex unprotected LM bifurcation lesions. As compared to DKC, rTAP reduced procedural time and improved side branch ostial expansion (8). The current study aims to compare the outcomes, as assessed by OCT, of both techniques at the intermediate term, i.e., 6-months follow-up.

The primary endpoint that we sought to examine is the serial change in the side branch ostial area in the 2 groups. No significant difference was detected in that respect between the 2 study arms (Table 3). Despite that a significantly better SB ostial stent expansion was achieved in rTAP vs. DKC at baseline, both groups had a similar ostial stent area at the end of the procedure (median >5 mm^2^ in both groups) (8). It is also crucial to note that a follow-up duration of 6 months does not cover the typical time-frame for restenosis in drug-eluting stents, and thus it is critical to observe how this plays out on the longer-term. Different authors have previously pointed out that multiple factors can influence such serial change, including features assessed by 3D-OCT: the SB jailing configuration by the MB stent struts, the site of crossing through the MB stent struts into the SB to perform the KBI (11, 12). These aspects warrant examination in further studies with 3D-OCT reconstruction.

From another perspective, stent strut apposition, tissue coverage, and the extent of neointimal growth are key elements to evaluate in the follow-up of different stenting techniques. The higher percentage of malapposed struts shown by follow-up OCT (including in part SB-ostium-jailing struts) in rTAP compared to DKC, albeit not reaching statistical significance, was not observed immediately after implantation. This can signify a higher incidence of late acquired malapposition. However, since the same stent type and similar deployment pressures were used in the two groups, the mechanisms of this phenomenon are unclear, but it might be hypothesized that the repeated high-pressure inflations might have resulted in more extensive vascular trauma. A more plausible explanation though is that the numerically fewer major malappositions detected at baseline in the DKC group (8), aided neointimal bridging of lesser degrees of malapposition (13–15) in this group as opposed to rTAP.

This tendency for a higher percentage of malapposed struts raises concern because of the unsettled relationship with stent thrombosis. However, it is important to note that the percentage of uncovered stent struts was not different between the 2 study groups, with the caveat that it can be difficult to discern neointima from fibrin depositions on malapposed struts by OCT. Interestingly, none of the patients in the 2 study arms had in-stent thrombotic events, or any kind of MI so far. However, as patients emerge from the protective umbrella of DAPT beyond 1 year, and should this malapposition remain unbridged, there is a higher theoretical risk of late and very late stent thrombosis.

On the other hand, the trend for larger neointimal area relative to the stent area in DKC could be explained by the repeated kissing, which creates more arterial wall stress and trauma, as previously shown by computer simulation, which may in turn trigger an excessive healing response (16). Another interesting finding was the significantly smaller luminal areas in the main branch distal to the bifurcation in DKC, associated with a tendency for smaller stent area, and larger neointimal area relative to the stent area, despite no difference in the used stent or post dilation-balloon diameters. This could be accounted for by the higher burden of calcification (as qualitatively judged on angiography—Supplementary Table S1) in the DKC group, precluding the parent artery stent from expanding to the same extent as in the rTAP group.

Although the current study was not powered to detect differences in clinical outcomes, it is worth noting that both groups showed low rates of target vessel failure which is reassuring from a safety standpoint.

### 4.1. Limitations

This was a small single-center study performed in a real-life setting, and the rate of loss to invasive follow-up was unfortunately relatively high. Further, the scope of the study was to investigate mid-term OCT parameters and not clinical outcomes, which would have required a much larger sample size.

A number of iterations of the protocol for DK-crush have been published, and we used the one proposed in the EBC consensus of 2018. This limitation obviously applies to any interventional study.

The current study involves only an intermediate-term follow-up. Given that in-stent restenosis typically needs more time to develop in drug-eluting stents, and that patients are still protected by DAPT at 6-months, longer-term follow-up is still needed to observe the impact of the OCT-detected differences on the clinical outcomes in the two study groups.

## 5. Conclusions

At 6-months, OCT showed a comparable change in the SB ostial area (primary endpoint) in rTAP vs. DKC. There were non-statistically significant trends for smaller luminal areas in the confluence polygon and the distal parent vessel, and larger neointimal areas relative to the stent areas, in DKC, as opposed to more malapposed stent struts in rTAP. The clinical impact of such signals is yet to be seen on longer term follow-up, as well as in other studies adequately powered to examine clinical endpoints.

## Data Availability

The raw data supporting the conclusions of this article will be made available by the authors, without undue reservation.
