# Supplementary material for: Six-months clinical and intracoronary imaging follow-up after reverse T and protrusion or double-kissing and crush stenting for the treatment of complex left main bifurcation lesions
Source: Front Cardiovasc Med. 2023 Apr 27;10:1153652. doi: 10.3389/fcvm.2023.1153652 (PMC10174439; doi:10.3389/fcvm.2023.1153652)
Supplement: Supplementary file 1 [file Table1.docx]

Supplementary Material

Six-months clinical and intracoronary imaging follow-up after reverse T and protrusion or double-kissing and crush stenting for the treatment of complex left main bifurcation lesions

Amr EI Abouelnour, Maximilian Olschewski, Giulio Makmur, Helen Ullrich, Maike Knorr, Majid Ahoopai, Thomas Münzel, Tommaso Gori^*^

*** Correspondence:** Tommaso Gori: [tommaso.gori@unimedizin-mainz.de](mailto:tommaso.gori@unimedizin-mainz.de)

# Supplementary Figures and Tables

## Supplementary Tables

**Supplementary Table 1. Baseline clinical characteristics for patients with follow-up OCT.**

|  | DK crush | | Reverse TAP | |
| --- | --- | --- | --- | --- |
|  | **N/Median** | **IQR** | **N/ Median** | **IQR** |
| Patient (n) | 19 |  | 19 |  |
| Age (years) | 71 | 64.25 to 81.5 | 73 | 69.25 to 80.75 |
| Female sex (n) | 6 |  | 3 |  |
| Syntax score | 26 | 20.75 to 30.75 | 26 | 22.25 to 29 |
| BMI (Kg/m²) | 28.37 | 26.04 to 30.58 | 25.88 | 24.73 to 27.75 |
| Obesity (n) | 5 |  | 3 |  |
| Smoking (n) | 1 |  | 1 |  |
| Prior smoking (n) | 7 |  | 6 |  |
| Hyperlipidemia (n) | 12 |  | 11 |  |
| Total cholesterol | 149 | 130.75 to 189 | 149 | 129.5 to 197.5 |
| Triglycerides | 99 | 75.5 to 141.25 | 122.5 | 76.5 to 216 |
| Hypertension (n) | 14 |  | 18 |  |
| Diabetes (n) | 7 |  | 5 |  |
| Prior CABG (n) | 2 |  | 2 |  |
| Prior PCI (n) | 8 |  | 6 |  |
| Prior stroke/TIA (n) | 1 |  | 2 |  |
| Clinical presentation |  |  |  |  |
| Stable angina/silent ischemia (n) | 15 |  | 13 |  |
| Unstable angina (n) | 2 |  | 2 |  |
| NSTEMI (n) | 2 |  | 4 |  |
| Baseline CK (U/L) | 70 | 46.75 to 131.25 | 92 | 66.75 to 105.25 |
| Baseline troponin (pg/mL) | 8.8 | 6 to 23.28 | 10.2 | 2.9 to 38.1 |
| BNP (pg/mL) | 86 | 22.5 to 146.5 | 74.5 | 35.5 to 156 |
| Creatinine (mg/dL) | 1.01 | 0.88 to 1.17 | 0.96 | 0.86 to 1.28 |
| eGFR (ml/min/1.73 m²) | 69 | 60 to 83 | 73 | 52 to 83 |
| LVEF (%) | 55 | 52 to 60 | 55 | 47.75 to 55 |
| Heart rate (beats/min) | 70 | 58.5 to 75 | 71 | 62 to 87.5 |
| Diastolic blood pressure (mmHg) | 70 | 61.25 to 79.25 | 70 | 59.5 to 75 |
| Systolic blood pressure (mmHg) | 136 | 107.75 to 153.25 | 129 | 107.75 to 143.5 |
| Lesion characteristics |  |  |  |  |
| Medina 1.1.1 (n) | 18 |  | 19 |  |
| Medina 1.1.0 (n) | 1 |  | 0 |  |
| Medina 0.1.1 (n) | 0 |  | 0 |  |
| Calcification of MB (n) | 19 |  | 18 |  |
| Calcification of SB (n) | 18 |  | 18 |  |
| Lesion length >10mm (n) | 17 |  | 17 |  |
| Multiple bifurcations (n) | 2 |  | 3 |  |
| Severe calcification (n) | 16 |  | 11 |  |
| Bifurcation angle >70 or <45° (n) | 18 |  | 16 |  |

N: number; IQR: inter-quartile range; CABG: coronary artery bypass grafting; PCI: percutaneous coronary intervention; NSTEMI: non-ST-elevation myocardial infarction; CK: creatine kinase; BNP: Brain-type natriuretic peptide; eGFR: estimated glomerular filtration rate; MB: main branch; SB: side branch.

**Supplementary Table 2. Procedural details of the index procedure for patients with follow-up OCT.**

|  | DK Crush | | Reverse TAP | |
| --- | --- | --- | --- | --- |
|  | **N/Median** | **IQR** | **N/Median** | **IQR** |
| Total heparin (IU) | 7500 | 7500 to 10000 | 7500 | 5000 to 10000 |
| Radial access (n) | 19 |  | 19 |  |
| Procedural time (min) | 31.0 | 24.5 to 44.75 | 25.0 | 22.25 to 31.5 |
| Contrast (ml) | 190 | 155 to 268.8 | 210 | 131.3 to 245.3 |
| Rotablation (n) | 1 |  | 2 |  |
| GP IIb/IIIa (n) | 4 |  | 3 |  |
| Multiple PCIs (n) | 6 |  | 9 |  |
| Predilation balloon diameter MB (mm) | 3.0 | 3.0 to 3.375 | 3.0 | 3.0 to 3.0 |
| Predilation balloon diameter SB (mm) | 3.0 | 3.0 to 3.0 | 3.0 | 2.81 to 3.0 |
| Stent diameter MB (mm) | 3.5 | 3.0 to 3.5 | 3.5 | 3.125 to 3.5 |
| Stent diameter SB (mm) | 3.0 | 3.0 to 3.5 | 3.0 | 3.0 to 3.0 |
| Final kissing balloon diameter MB (mm) | 3.0 | 2.625 to 3.375 | 3.0 | 3.0 to 3.0 |
| Final kissing balloon pressure MB (atm) | 14.0 | 12 to 18 | 12.0 | 12.0 to 14.0 |
| Final kissing balloon diameter SB (mm) | 3.0 | 2.6 to 3.0 | 3.0 | 3.0 to 3.0 |
| Final kissing balloon pressure SB (atm) | 14.0 | 12.0 to 18 | 12.0 | 12.0 to 14.0 |
| Final POT balloon diameter (mm) | 4.0 | 3.5 to 4.0 | 4.0 | 4.0 to 4.5 |
| Therapy with anticoagulants (n) | 5 |  | 4 |  |
| DAPT type (n) |  |  |  |  |
| Clopidogrel | 2 |  | 4 |  |
| Ticagrelor | 11 |  | 10 |  |
| Prasugrel | 6 |  | 5 |  |
| Peak postprocedural troponin (ng/L) | 206 | 72.8 to 1017 | 334 | 52.5 to 568.5 |
| Post-OCT optimization (n) | 4 |  | 7 |  |

N: number; IQR: inter-quartile range; GP: glycoprotein; MB: main branch; SB: side branch
